# Prevalence of Preterm Birth Rate During COVID-19 Lockdown in a Tertiary Care Hospital, Riyadh

**DOI:** 10.7759/cureus.13634

**Published:** 2021-03-01

**Authors:** Roya Huseynova, Latifa Bin Mahmoud, Adli Abdelrahim, Morabet Al Hemaid, Muath S Almuhaini, Parameaswari P Jaganathan, Halima Career, Ogtay Huseynov

**Affiliations:** 1 Neonatal Intensive Care Unit, King Saud Medical City, Riyadh, SAU; 2 Pediatrics, King Saud Medical City, Riyadh, SAU; 3 Research and Innovation, King Saud Medical City, Riyadh, SAU; 4 Obstetrics and Gynecology, King Saud Medical City, Riyadh, SAU; 5 Medicine, Azerbaijan Medical University, Baku, AZE

**Keywords:** covid-19, gestation age, pandemic, preterm birth, preterm infants, sars-cov-2 in pediatric patients, birth rate, sars-cov-2

## Abstract

Background

On March 3, 2020, the first case of coronavirus disease (COVID-19) was reported by the Ministry of Health, Kingdom of Saudi Arabia. Within days, the government confirmed more cases and adopted lockdown measures with travel restrictions from March to June 2020. A distinctive coronavirus was isolated from 190,823 patients by June 30. The pandemic resulted in a significant risk to public health. The study aimed to evaluate the impact of COVID-19 lockdown on the rate of premature births.

Method

In this cross-sectional study, we observed premature births at the Neonatal Intensive Care Unit (NICU). The study site is a 1,500-bed teaching hospital, with around 4,500 annual deliveries, 70 beds in level II and level III, and tertiary care NICU. We compared the birth rates among preterm infants between March 1 to June 30, 2017-2019, to the similar calendar months of 2020. Information on nationality, gestational age, and maternal conditions were collected from the medical records. We used the Poisson regression model to assess the preterm birth rate's temporal trends before lockdown versus during lockdown.

Results

Among 7,226 total live neonates, we recorded 1,320 preterm infants during the study period of 2017-2020. The preterm birth rate per 1,000 live births during lockdown showed a 23% drop in the overall preterm birth rate with Prevented Fraction of 36% in extremely preterm (<28 weeks gestational age) births and 26% in moderate/late premature (32 weeks to 36 weeks + 6 days gestational age) births. The estimated preterm birth rate among the Saudi expats (15.11/1,000 live births) showed an increased tendency compared to Saudi nationals (odds ratio [OR]=1.07; 95% CI: 0.75-1.52) and was statistically not significant during the strict lockdown.

Conclusion

There was a significant reduction in the birth rate of extremely preterm and moderate/late preterm infants during lockdown when compared to the preceding three years. A national dataset is required to evaluate the extent of lockdown's impact on the preterm birth rate.

## Introduction

Preterm birth (PTB) is defined as a live birth before completing 37 weeks of pregnancy. PTB across nations remains a leading cause of infant mortality and morbidity, which adds significant challenges to their health and quality of life compared to term neonates [1].

The majority of PTB is spontaneous preterm birth [2]. Other causes of preterm delivery include intrauterine infections, multiple gestation, hormonal imbalance, chronic conditions like diabetes, high blood pressure, and genetic factors; however, no cause is often identified. Ananth et al. reported an increased tendency of preterm birth due to medical indications (iatrogenic), mostly due to obstetric intervention [3]. Other factors associated with an increase in preterm birth rate included deviations in older maternal age and frequency of artificially conceived multiple pregnancies [4].

It remains unclear which intervention is best to prevent spontaneous preterm births in women with risk factors [5]. The majority of reviews on PTB causes have focused on ascending infections and genetic causes, the effectiveness of progesterone for the prevention of PTB, and the association between work and behavior on PTB [4-6]. Several factors, such as stress, social, or maternal anxiety, have been linked to premature deliveries. The potential influence of biological, psychological, and environmental factors could cumulatively influence and modify the PTB incidence [6].

Wheeler et al. reported positive benefits from family support on reducing PTB rates [6]. Also, the daily involvement of pregnant women's partners is considered another positive factor in reducing the incidence of preterm deliveries as women supported by their partners reported to have a better advantage on their general wellbeing [7]. Many other studies have been conducted on socio-economic factors and their effect on preterm labor, with their assessment based mainly on occupation, income, and education. Job opportunities, including professional careers, physical working environment, and working hours, have been considered to assess their impact on preterm delivery. Working environment rather than the time spent at work was reported to have an impact on preterm births. Those working in healthcare or school environments, managerial jobs, or clerical positions would have a lower PTB incidence than manufacturing jobs, sales, or service [8].

Saudi Arabia's Ministry of Health (MOH) worked to achieve the concepts related to providing recovery, harmony, and sustainability to the health system. It launched educational campaigns for the public to follow strict hand-hygiene measures, social distancing, and adherence to MOH recommendations and as a collective effort to reduce the transmission of coronavirus disease (COVID-19). A nationwide curfew was adopted from March to June 2020 with the temporary closing of shops, colleges, schools, and all non-essential institutions. The lockdown occurred in two phases: complete lockdown in phase 1 with restricted mobility and travel by air, land, and sea for 24 hours, followed by 8 and 5 hours in phase 2 partial quarantine [9]. The limited overall vehicular traffic and commuting to and from work locations reduced the chances of car crashes involving pregnant women and traffic-related stresses. The lockdown period allowed an exceptional opportunity for us to study the effects of lockdown as the critical determinant in restoring the overall health of the 'intrauterine habitat' and whether it would influence the continuation of fetal life.

## Materials and methods

We conducted this cross-sectional study at the Neonatal Intensive Care Unit (NICU), Public Sector Children Hospital - King Saud Medical City (KSMC), covering four referral hospitals and 10 primary healthcare centers in Saudi Arabia. The inclusion criteria were all live infants born at KSMC between March 1 till June 30, 2017-2020, and the exclusion criteria were abortions, stillbirths, and intrauterine fetal death (IUFD). We collected data on live births, PTB, gestational age in weeks, and maternal details, including maternal conditions and nationality (Saudi national/Saudi expat) for the study period from the hospital logbook of delivery. We categorized the 1,320 preterm infants according to gestational age at birth as extremely preterm (24-27 weeks + 6 days), very preterm (28-31 weeks + 6 days), and moderate to late preterm (32-36 weeks + 6 days).

The Institutional Review Board approved the present study as per the National Bioethics rules and regulations (H1RI-30-Jun20-01). The data was analyzed using SPSS 25.0 (IBM SPSS Statistics for Windows, Version 25.0. Armonk, NY: IBM Corp). The variables under study were categorical and presented as frequency and percentage. Here, we used the One-Sample Test for Binomial Proportion, Chi-Square, Normal-Theory Method, Fisher's Exact (Clopper-Pearson), and 95% confidence interval (CI) for the statistical significance. The Poisson regression model was derived to assess the preterm birth rates' temporal trends per 1,000 live births over 16 months. We compared these estimates for March-June 2020 with the 95% Wald confidence interval and risk ratio analysis with the previous years. 

## Results

**Figure 1:**
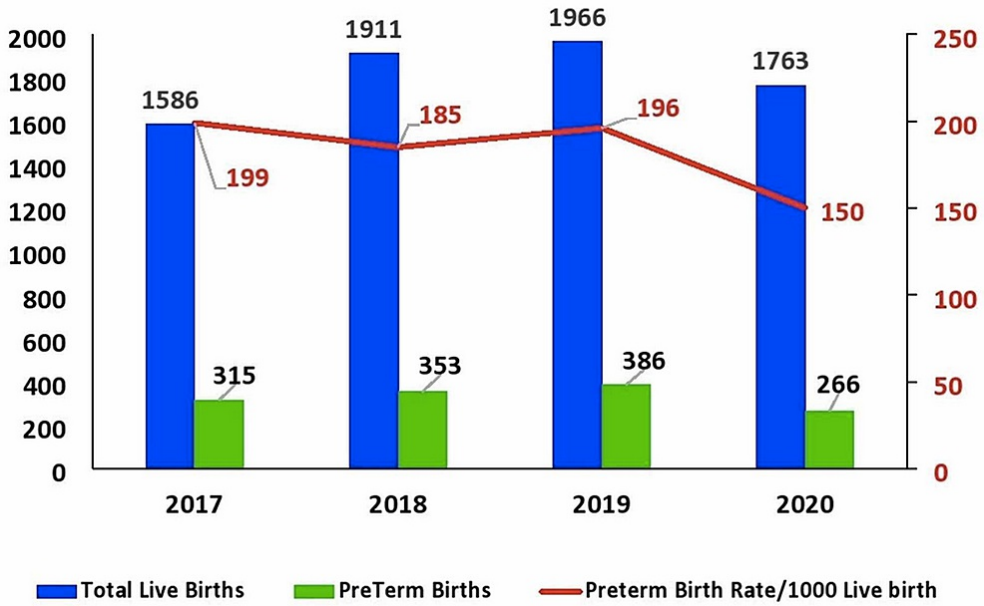
Preterm birth rate *Blue vertical line indicates the total number of live births from March 1 to June 30. *Green vertical line indicates the number of preterm births from March 1 to June 30. *Red horizontal line indicates the preterm birth rate per 1,000 live births from March 1 to June 30.

Our study identified 7,226 live births during the study period from March 1 to June 30 of 2017-2020, with 3,832 (49.6%) male and 3,894 (50.4%) female infants. We estimated the overall PTB rate per 1,000 live births for the pre-lockdown period (March-June of 2017-2019) and the nationwide lockdown (March-June of 2020) as exhibited in Figure 1.

This figure established an increase of 19% in total live births between 2017 to 2019, with a 10% decrease from 2019 to 2020. PTB rate per 1,000 live births showed a 7% decline from 2017 to 2018 and a 23% decrease during the COVID-19 lockdown period in 2020. We also analyzed the data based on ethnicity into two groups - Saudi nationals and Saudi expats. The PTB rate was more among Saudi expats than Saudi nationals, with significant differences during March and June of 2020 (Table 1).

**Table 1:** Preterm birth rate per 1,000 live births among the Saudi nationals and Saudi expats (March 1 - June 30, 2017-2020) *Statistically significant at 5% level

Year	Month	Total live births		Preterm birth rate/1,000 live births		Chi-square statistic
		Saudi Nationals	Saudi Expats	Saudi Nationals	Saudi Expats	(P-value)
2017	Mar	266	81	135.3	283.9	0.0019*
2017	Apr	235	64	106.4	187.5	0.0812
2017	May	402	82	111.9	292.7	0.0001*
2017	June	353	103	93.5	223.3	0.0004*
2018	Mar	402	93	72.1	322.6	0.0001*
2018	Apr	278	93	97.1	75.3	0.5287
2018	May	439	85	14.4	235.2	0.0001*
2018	June	412	109	87.4	192.7	0.0018*
2019	Mar	398	95	82.9	126.3	0.1874
2019	Apr	360	108	12.2	166.7	0.0001*
2019	May	369	121	81.3	132.2	0.0961
2019	June	396	119	78.3	151.2	0.0176*
2020	Mar	299	97	107	237.1	0.0013*
2020	Apr	276	114	43.5	87.7	0.0857
2020	May	328	92	85.4	130.4	0.1944
2020	June	443	114	92.6	157.9	0.0436*

Poisson regression analysis for 2017-2020 measured a temporal trend in PTB rates with Wald χ2 = 46.08 (P=0.000). We observed a reduction of the PTB rate per 1,000 live births in both populations, with more significant among Saudi expats (decrease of 4 preterm births/1,000 live birth versus 2/1,000 live births among Saudi nationals) based on the Chi-square test for proportions; however, it was not statistically significant (Table 2).

**Table 2:** Comparison between preterm birth rate before (2017-2019) and during COVID-19 lockdown (2020) among the Saudi population

Saudi Population	Preterm birth rate/1,000 Live births		Chi-square statistic	Odds Ratio
	(Mar-June)		(P-value)	(95% Confidence Interval)
	2017-2019	2020		
Saudi Nationals	10.02	8.39	0.272 (0.602)	-
Saudi Expats	18.62	15.11	0.412 (0.521)	1.07 (0.75 – 1.52)

Table 3 shows the PTB for the combined years 2017-2019 compared with 2020 for each gestational category. The Chi-square test for proportions shows a statistically significant decrease in the two groups of extremely preterms and moderate/late preterms during the lockdown. Considering the population >37 weeks as the reference category, we estimated the Relative Risk for the three gestational age groups. We observed 36.2% and 26.34% prevented fraction of preterm births during COVID-19 lockdown in extremely preterm and moderate/late preterms births, respectively.

**Table 3:** Prevented Fraction of preterm births in COVID-19 lockdown (March 1 - June 30) *Statistically significant at 5% level

Gestational age (weeks)	Total live births	Preterm births		Chi-square statistic	Relative Risk	Prevented Fraction of preterm births due to COVID-19 lockdown
	2017-2020	2017-2019	2020	(P-value)		
< 28	130	107	23	3.956 (0.047)*	63.80%	36.20%
28-31+^6^	215	160	55	0.006 (0.938)	101.20%	-1.20%
32-36+^6^	975	787	188	16.647(0.0004)*	73.66%	26.34%
>37	5906	4409	1497	-	-	-
Total	7226	5463	1763			

We compared the calculated PTB rate for each preceding year (2017, 2018, 2019) with the lockdown period (2020) and tested for its significance (Table 4). The preterm birth rate drop ranged from 2.5% to 42.5% in the years 2017-2020 in the extremely premature group, and an escalation of 36% was observed with a further 21% decay in 2019 to 17% incline in 2020 in the very premature group. We observed an increasing trend from 12% to 21% in 2018 and 2019; however, there was a rapid incline to 36% among the 32-36 weeks + 6 days preterm birth rates during the COVID-19 lockdown. Even though there was a reducing tendency among the birth rates between these four consecutive years, we did not identify a statistically significant reduction during the COVID-19 lockdown (Table 4). However, there was a significant difference in threatened preterm labor and multiple pregnancies between 2020 and 2017-2019 when we compared based on potential causes (Table5).

**Table 4:** Preterm births for gestational age (March 1-June 30, 2017-2020) *Statistically significant at 5% level GA: gestational age

Year	Total Preterm Births	GA:<28 wks	GA: 28-31+^6^ wks	GA: 32-36+^6^ wks	Total live births
	(birth rate/1000 live births)	(birth rate/1000 live births)	(birth rate/1000 live births)	(birth rate/1000 live births)	
2017	315 (198)	40 (25.2)	42 (26.5)	233 (146.9)	1586
P-value	0.1391	0.7456	0.8921	0.2205	-
2018	353 (185)	28 (14.6)	66 (34.5)	259 (135.5)	1911
P-value	0.2658	0.9616	0.9197	0.3595	-
2019	386 (196)	39 (19.8)	52 (26.4)	295 (150)	1966
P-value	0.1399	0.8439	0.8827	0.1714	-
2020	266 (151)	23 (13)	55 (31.2)	188 (106.6)	1763

**Table 5:** Causes of preterm births (March 1-June 30, 2017-2020) *Statistically significant at 5% level

	2017-2019	2020	Average (2017-2019) (%)	Average 2020 (%)	P-value	95% CI
Hypertensive disorders of pregnancy	71	13	17.5	12.7	0.3109	4.7489% to 13.6616%
Premature rupture of membrane	37	11	8.7	10.8	0.5864	5.4299% to 10.4506%
Fetal distress	36	15	8.7	14.7	0.1475	2.1357% to 14.9332%
Multiple pregnancies	24	0	5.8	0	0.0136*	1.1923% to 11.0497%
Oligo-/polyhydramnios	13	0	2.9	0	0.0835	1.1380% to 7.2393%
Uterine scar	45	12	10.9	11.7	0.8468	7.1924% to 9.5391%
Malpresentation	55	13	13.1	12.7	0.9275	8.7184% to 8.8153%
Antepartum hemorrhage	62	23	14.6	22.5	0.1164	1.9266% to 18.1832%
Others	8	1	1.5	0.9	0.6796	3.8478% to 4.4017%
Threatened preterm	31	4	7.3	1.1	0.0311*	0.3975% to 11.888%
Infectious disease	36	10	8.8	4.3	0.1897	2.7493% to 10.9862%

## Discussion

Our study, conducted in a tertiary hospital in Riyadh, Saudi Arabia, showed a significant reduction in preterm birth rates during quarantine in two groups, extremely premature and late preterm, compared to a control group of correlating calendar years from 2017-2019. However, we did not identify a statistically significant reduction in the overall PTB rate (Table 4).

Similar observations have been reported in multiple studies conducted in Ireland, Denmark, Australia, and the Netherlands with differences in the impacted age groups (extremely premature, very premature, moderate/late premature), which may imply an international reduction trend of PTB. Researchers from Denmark observed a 90% reduction in extremely premature neonates (OR 0.09, P=0.01) when compared to the previous five years [10]. In a preprint study, Australian researchers have also observed 29 per 1,000 moderate/late PTB versus an average of 64 per 1,000 births (OR 0.39) when comparing this age group to the previous seven years [11]. Similar findings from the Netherlands showed a reduction in the early period of lockdown (OR 0.77, P=0.0026) compared to the previous 10 years [12]. In Ireland, a 73% reduction in delivery of very low birth weight infants was observed compared to the last 20 years [13]. These findings could be related to strict lockdown measures implemented by these countries and shared mitigation measures such as public health awareness, hygiene, clean water, social distancing, reduced in-hospital visits, and travel restrictions. Chibber et al. reported a higher risk of preterm deliveries among the primigravida women following air traveling [14]. Reduced work-related stressors, proper sleep and nutrition, exercise, less exposure to tobacco and infections might be among the plausible contributing factors in lowering preterm deliveries in these reports [4,5]. However, similar strict measures have been adopted in California, USA, which showed no differences in PTB rates during lockdown nor differences in ethnic or racial groups [15]. Similarly, we did not find a statistically significant difference in our population in regards to ethnic groups. In contrast to our result, a recently published paper in Nepal described an increased PTB rate during quarantine [16], although none of the recorded pregnant women in the Nepal study were tested for COVID-19. A meta-analysis study and other reports observed the relation of COVID-19 and PTB [17-19]. 

The reduction we observed in our study can be unrelated to the event of lockdown but explained by the singleton pregnancy in our data during quarantine (P=0.0136) and decreased maternal disorders related to threatened preterm labor (P= 0.0311) (Table 5). Limitations to this study include the retrospective design, single-center setting, short frame, and we have not explored the specific acquired lifestyle modifications by the pregnant women during the lockdown. Another limitation of this study is that we did not assess the incidence of stillbirths, abortions, or IUFD during the study period nor the percentage of confirmed COVID-19 mothers in this study. Moreover, limited admission to our center at the beginning of the lockdown could have influenced this observed reduction.

## Conclusions

This study evaluates the effect of Saudi national lockdown on the rate of preterm births. The significant reduction observed in extremely premature and moderate/late premature birth rates anticipates a reduction of morbidity and mortality as well as reduced burdens on families and medical staff, especially in regards to the extremely premature group. Similar reports from studies conducted in Ireland, Denmark, and Australia during the lockdown period would hint at valuable future research opportunities that can aim to discover modifiable PTB risks. Further studies on lifestyle, behavioral, physical, and psychosocial modifications adopted by pregnant women during the lockdown would illustrate a better understanding and aid in devising new guidelines and preventative measures for future pregnancies. 
